# Male-specific long non-coding RNA testis-specific transcript, Y-linked 15 promotes gastric cancer cell growth by regulating Wnt family member 1/β-catenin signaling by sponging microRNA let-7a-5p

**DOI:** 10.1080/21655979.2022.2053814

**Published:** 2022-03-25

**Authors:** XiaoYing Zheng, BingJun Peng, XinChun Wu, JunLing Ye, HaiYun Zhao, YanJun Li, RuiHui Chen, Xue Gong, HaiYan Zhang, XinJian Guo

**Affiliations:** a Department of Pathology, Affiliated Hospital of Qinghai University, Xining, China; b Department of Medical Imaging Center, Affiliated Hospital of Qinghai University, Xining, China; c Fourth Department of Internal Medicine, Qianxi County People’s Hospital, Tangshan, China; d Department of Pathology, Menyuan Hui Autonomous County traditional Chinese Medicine Hospital, Qinghai, China

**Keywords:** TTTY15, let-7a-5p, Wnt/β-catenin pathway, gastric cancer

## Abstract

The present study is aimed to investigate the regulatory effects and related mechanism of long non-coding RNA testis-specific transcript, Y-linked 15 (TTTY15) in gastric carcinoma (GC) cell proliferation, migration, invasion, apoptosis and epithelial–mesenchymal transition (EMT). TTTY15 expression in GC tissue samples and cells was detected by quantitative real-time PCR (qRT-PCR), and the correlation between TTTY15 expression and GC clinicopathological indicators was analyzed. Cell counting kit-8 (CCK-8), BrdU, flow cytometry and Transwell assays were performed for detecting GC cell proliferation, migration, invasion and apoptosis. Western blot was performed for detecting the expressions of EMT-associated proteins (N-cadherin and E-cadherin), Wnt family member 1 (Wnt1) protein and β-catenin protein. Bioinformatics analysis was conducted to predict, and RNA immunoprecipitation (RIP) assay and dual-luciferase reporter gene assay were performed to verify the targeted relationships of microRNA let-7a-5p (let-7a-5p) with TTTY15 and Wnt1 mRNA 3'UTR. It was found that TTTY15 expression was significantly up-regulated in GC tissues and cells, and was associated with advanced TNM stage and poor tumor differentiation. TTTY15 overexpression promoted GC cell proliferation, migration and invasion, the expressions of N-cadherin, Wnt1 and β-catenin protein, and inhibited the apoptosis and E-cadherin expression, while knocking down TTTY15 had the opposite effects. TTTY15 directly targeted let-7a-5p and negatively regulated its expression. Wnt1 was the target gene of let-7a-5p, and TTTY15 could indirectly and positively regulate Wnt1 expression. In conclusion, TTTY15 promotes GC progression, by regulating the let-7a-5p/Wnt1 axis to activate the Wnt/β-catenin pathway.

## Graphical Abstract

**Figure:**
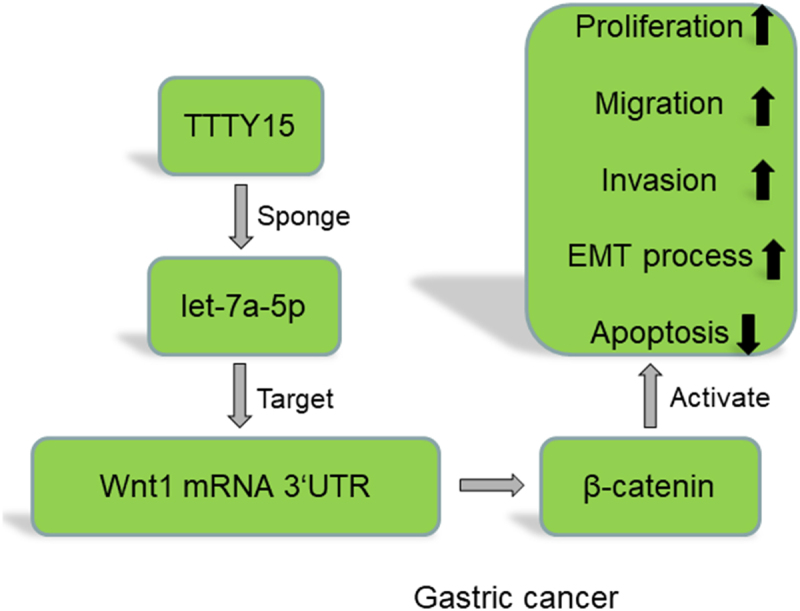

## 1. Introduction

Gastric cancer (GC) is a malignancy originating from the gastric mucosa epithelium [1]. The current methods for GC treatment mainly include surgical resection, radiotherapy and chemotherapy [2,3]. Due to the fact that the early symptoms are insidious, most patients are already in the advanced stage when they are diagnosed with GC, and have missed the opportunity of a radical cure through surgery [4]. Additionally, the epidemiology of GC is different in men and women, and it is more prevalent in the male population and carries a worse prognosis, and the incidence of GC in men is almost twice as high as that in women [5]. Nonetheless, the molecular mechanism leading to the difference in the incidence of GC in different genders has not been elucidated.

Known as a type of non-coding RNA (ncRNA) with more than 200 *nt* in length, long non-coding RNAs (lncRNAs) have no protein-coding function [6,7]. There are increasing studies suggesting that lncRNAs are closely linked with the pathogenesis of human diseases, including cancer [8]. LncRNA testis-specific transcript, Y-linked 15 (TTTY15)’s coding sequence is located on the Y chromosome. Previous research has suggested that in most prostate carcinoma (PCa) patients’ tumorous tissues, TTTY15 is the most up-regulated lncRNA on the Y chromosome; TTTY15 overexpression can promote PCa cell proliferation and metastasis [9]. Additionally, our previous study and other groups’ studies suggest that TTTY15 is involved in regulating the progression of esophageal squamous cell carcinoma and colorectal cancer [^9–11^]. Nevertheless, the biological functions and mechanism of TTTY15 in GC remain unclarified.

Wnt/β-catenin signaling pathway is the ‘star pathway’ in cancer research [12,13]. In GC, the activation of Wnt/β-catenin signaling regulates the biological behaviors of cancer cells. For instance, tissue transglutaminase-1 (TGM1) can activate the Wnt/β-catenin pathway to enhance the stemness of cancer stem cell (CSC) and chemotherapy resistance of GC cells [14]. In the present work, bioinformatics analysis showed that TTTY15 could probably directly target microRNA let-7a-5p (let-7a-5p), and let-7a-5p could probably target Wnt family member 1 (Wnt1) 3'UTR. We hypothesized that TTTY15 had the potential to be the diagnostic biomarker and therapeutic target for GC. In this work, we investigated the biological function and mechanism of TTTY15 in GC progression. Herein, we report that TTTY15 promotes GC cell growth, migration and invasion, and inhibits apoptosis through targeting the let-7a-5p/Wnt1 axis.

## 2. Materials and methods

### 2.1. Clinical tissue sample collection

The present study was endorsed by the Ethics Committee of the Affiliated Hospital of Qinghai University, and all participants signed an informed consent. We selected 59 male GC patients who were admitted to the Affiliated Hospital of Qinghai University from May 2017 to May 2020, and collected the surgically resected cancerous tissues and para-cancerous tissues. After the surgical resection, the tissues were kept in the refrigerator (−80°C).

### 2.2. Cell culture [15]

The gastric mucosal epithelial cell line GES-1 was obtained from China Center for Type Culture Collection (CCTCC, Wuhan, China), and the GC cell lines (NCI-N87, SNU-1 and KATO III) were obtained from American Type Culture Collection (ATCC) (Manassas, VA, USA). All cells were cultured in Dulbecco’s Modified Eagle Medium (DMEM, Invitrogen, Carlsbad, CA, USA) with 100 U/ml penicillin (Invitrogen, Carlsbad, CA, USA) + 0.1 mg/ml streptomycin (Invitrogen, Carlsbad, CA, USA) and 10% fetal bovine serum (FBS, Invitrogen, Carlsbad, CA, USA). The cells were placed in the incubator under conditions of 95% humidity and 5% CO_2_ at 37°C. When the cells were in the logarithmic growth phase, they were harvested for subsequent experiments.

### 2.3. Cell transfection [16]

NCI-N87 and SUN-1 cells were transferred at 1 × 10^6^ cells/ml into 60-mm culture plates and cultured, and the cells were transfected after 24 h. The pcDNA empty vector (oe-NC), pcDNA-TTTY15 overexpression plasmid (oe-TTTY15), small interfering RNA targeting TTTY15 (si-TTTY15-1 and si-TTTY15-2) and the negative controls (si-NC), let-7a-5p mimics and its control (mimics NC), and let-7a-5p inhibitors and its control (inhibitors NC) were purchased from RiboBio Co., Ltd. (Guangzhou, China). The transfection was conducted using Lipofectamine® 2000 kit (Invitrogen, Carlsbad, CA, USA) following the manufacturer’s instruction. After 24 h of transfection, Western blot and quantitative real-time PCR (qRT-PCR) were utilized to detect the transfection efficiency.

### 2.4. qRT-PCR [17]

TRIzol reagent (Invitrogen, Carlsbad, CA, USA) was utilized for total RNA extraction from tissues or cultured cells. The Reverse Transcription Kit (Takara, Dalian, China) was adopted to reverse-transcribe RNA into cDNA. qRT-PCR was performed using a SYBR Green PCR kit (Takara, Dalian, China) on an Applied Biosystems 7300 Real-Time PCR System (Thermo Fisher Scientific, Inc., Foster City, CA, USA). With U6 as the internal control for let-7a-5p, and GAPDH as the internal control for TTTY15 and Wnt1, the relative expression was calculated by the 2^−ΔΔCt^ method. The primer sequences are as follows (F for forward; R for reverse). TTTY15 primer sequence: F, 5’-TCTATGACCTGGAAGC-3’; R, 5’-ATCTGATGGAACCCTA-3’. Let-7a-5p primer sequence: F, 5’-GGTGAGGTAGTAGGTTGTATAGTT-3’; R, 5’-CTCGCTTCGGCAGCACATATA-3’. U6 primer sequence: F, 5’-CTCGCTTCGGCAGCACA-3’; R, 5’-AACGCTTCACGAATTTGCGT-3’. Wnt1 primer sequence: F, 5’-TGGCTGGGTTTCTGCTACG-3’; R, 5’-CCCGGATTTTGGCGTATC-3’. GAPDH primer sequence: F, 5’-AGCCACATCGCTCAGACAC-3’; R, 5’-GCCCAATACGACCAAATCC-3’.

### 2.5. Survival analysis [18]

Bioinformatics analysis was conducted using KM-plotter database (http://kmplot.com/analysis/) to analyze the correlation between TTTY15 expression and overall survival (OS) and disease-free survival (DFS) of GC patients.

### 2.6. Cell counting kit-8 (CCK-8) assay [19]

NCI-N87 and SUN-1 cells were transferred into 96-well plates (2 × 10^3^ cells/well). After the cells were attached to the wall, the samples were added with 90 μL of medium and 10 μL of CCK-8 solution (Dojindo Molecular Technologies, Japan). After 2 h of incubation, the absorbance (optical density, OD) value at 450 nm wavelength of each well was measured with a microplate reader and recorded. The OD values of the cells at 12, 24, 36, 48 and 72 h were determined, and the cell growth curve was drawn with time as the x-coordinate and OD value as the y-coordinate.

### 2.7. BrdU assay [20]

NCI-N87 and SUN-1 cells were transferred into 24-well plates. When the cells reached logarithmic growth phase, each well was added with 200 μL of 5 μmol/L BrdU working solution (Beyotime Biotechnology, Shanghai, China). The cells were then incubated for 2 h in the incubator and then washed with phosphate buffer saline (PBS). Subsequently, after the fixation of the cells with paraformaldehyde, the cells were incubated with 2 mg/ml glycine for 5 min. Next, each well was added with 100 μL of PBS containing 0.5% TritonX-100, and the cells were decolorized for 10 min on a shaker, followed by PBS washing. Next, the cells were incubated with 1× Hoechst 33342 DNA staining solution at room temperature in the dark for 20 min. After PBS washing, the cells were photographed and counted under a fluorescence microscope.

### 2.8. Flow cytometry [21]

The AnnexinV-FITC/PI cell apoptosis double staining kit (BD Biosciences, New Jersey, USA) was employed for detecting cell apoptosis. The NCI-N87 and SUN-1 cells during logarithmic growth were collected, and re-suspended in binding buffer (100 μL per sample). Next, 5 μL of AnnexinV-FITC staining solution was supplemented, and the mixture was mixed gently and incubated in the dark at room temperature for 15 min. Subsequently, with the addition of 10 μL of PI staining solution, they were mixed gently again, and placed in ice to incubate for 5 min in the dark. Ultimately, flow cytometry was performed within 30 min, and the apoptosis index is calculated as follows: [apoptosis index (%) = AnnexinV(+)PI(-)(%) + AnnexinV(+)PI(+)(%)].

### 2.9. Transwell assays [22]

For cell migration assay, NCI-N87 and SUN-1 cells were re-suspended in serum-free medium, and 100 μL of cell suspension was added to the upper compartment of the Transwell chamber (Corning Incorporated, Corning, NY, USA), and the lower compartment of the Transwell chamber was added with 500 μL of complete medium containing 10% FBS. The cells were cultured for 24 h. Subsequently, the chamber was washed twice with PBS, and cotton swabs were utilized to gently wipe off the non-migrated cells in the top compartment. Then the remaining cells were fixed with 4% paraformaldehyde for 15 min. Then, they were washed 3 times with PBS, and stained for 10 min with 0.1% crystal violet. Eventually, the cells were washed with PBS again, and in five randomly selected fields, the stained cells were counted. For cell invasion assay, except that the top chamber was pre-coated with Matrigel, the rest of the steps were the same as in the migration assay.

### 2.10. Dual luciferase reporter gene assay [23]

TTTY15 and Wnt1 3'UTR sequences were cloned into the luciferase reporter plasmid pmirGLO (Promega, Madison, WI, USA). Then, the wild type (WT) and mutant type (MUT) luciferase reporter pmirGLO-TTTY15-WT (WT TTTY15), pmirGLO-Wnt1-WT (WT Wnt1), pmirGLO-TTTY15-MUT (MUT TTTY15) and pmirGLO-Wnt1-MUT (MUT Wnt1) were constructed. Subsequently, let-7a-5p mimics (or control mimics) and WT TTTY15 (MUT TTTY15), and let-7a-5p mimics (or control mimics) and WT Wnt1 (MUT Wnt1) were co-transfected into GC cells, and 48 h later, the luciferase activity was detected on a dual-luciferase reporter assay system (Promega, Madison, WI, USA) in compliance with the protocols.

### 2.11. RNA immunoprecipitation (RIP) assay [24]

The Magna RIP™ RNA binding protein immunoprecipitation kit (Millipore, Billerica, MA, USA) was used to perform RIP experiment. The GC cells were lysed by RIP lysis buffer, and then were incubated with anti-Argonaute-2 (Ago2) antibody coupled with magnetic beads. Anti-IgG antibody was defined as the negative control. Then the immunoprecipitated complex was treated with proteinase k, and subsequently the RNA was isolated, and then TTTY15 and let-7a-5p level in the precipitates was quantified by qRT-PCR.

### 2.12. Western blot [25]

The GC cells were lysed employing RIPA lysis buffer (Beyotime Biotechnology, Shanghai, China) containing protease inhibitors. After high-speed centrifugation, the supernatant was collected and heated in a 100°C water bath for 10 min to denature the protein. The proteins were separated by sodium dodecyl sulfate-polyacrylamide gel electrophoresis (SDS-PAGE) after protein quantification by the BCA protein assay kit (Beyotime Biotechnology, Shanghai, China), and then transferred onto the PVDF membrane. Then, the membranes were washed with tris buffered saline tween (TBST) solution, and they were incubated overnight with rabbit anti-N-cadherin antibody (Abcam, ab76011, 1:500), rabbit anti-E-cadherin antibody (Abcam, ab212059, 1:500), rabbit anti-Wnt1 antibody (Abcam, ab15251, 1:500), rabbit anti-β-catenin (Abcam, ab32572, 1:500), rabbit anti-β-actin antibody (Abcam, ab8227, 1:500) at 4°C. After rinsing the PVDF membranes with TBST solution again, they were incubated with goat anti-rabbit IgG H&L (HRP) (Abcam, ab150077, 1:1000) at room temperature for 1 h. After rinsing the membranes with TBST again, the protein bands were developed by the hyper-sensitive ECL kit (Beyotime Biotechnology, Shanghai, China).

### 2.13. Statistical analysis [26]

The analysis tool for the experimental data was SPSS 20.0 software (SPSS Inc., Chicago, IL, USA). Mean ± standard deviation was the expression form of all measurement data. The comparison between two groups was conducted using *t*-test, and one-way analysis of variance was conducted for the comparison among three or more groups; the skewed data were compared with non-parametric test. The enumeration data were represented in contingency table and analyzed by χ^2^ test. A difference was statistically significant when *P* < 0.05.

## 3. Results

We hypothesized that TTTY15 could promote the progression of GC. Gain-of-function and loss-of-function models were established, and it was revealed that TTTY15 could regulate the malignant biological behaviors of GC cells. Additionally, it was demonstrated that TTTY15 directly targeted let-7a-5p/Wnt1 axis, and the overexpression of let-7a-5p reversed the effects of TTTY15 on proliferation, migration, invasion, epithelial-mesenchymal transition (EMT) and apoptosis of GC cells.

### 3.1. TTTY15 is up-regulated in GC

**Figure 1.**
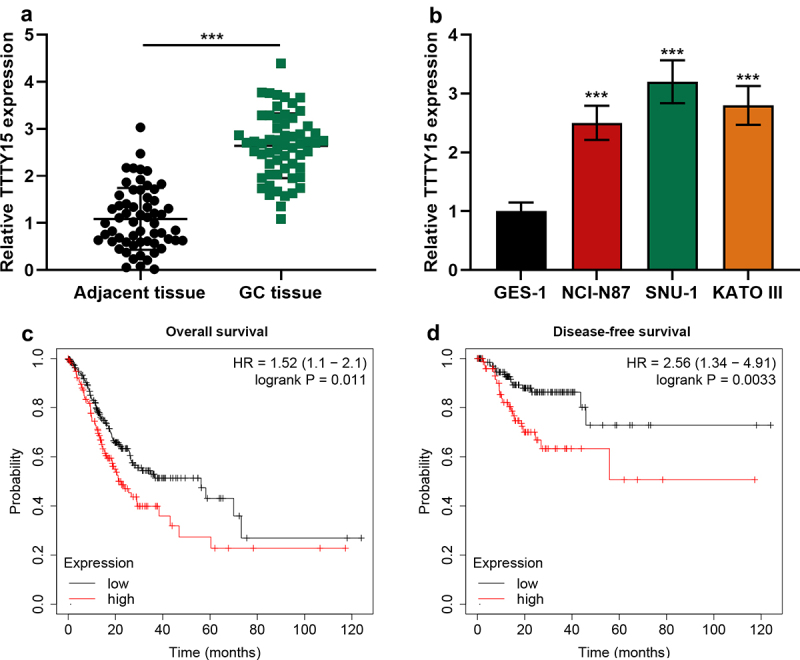
The expression characteristics of TTTY15 in GC. (a) TTTY15 expression in GC tissues and para-tumorous tissues was detected by qRT-PCR. (b) TTTY15 expression in GC cell lines (NCI-N87, SNU-1 and KATO III) and GES-1 cells was detected by qRT-PCR. (c and d) Kaplan-Meier Plotter database was applied for analyzing the association between TTTY15 expression and the GC patient’s survival time. All of the experiments were performed in triplicate. *** *P* < 0.001.

Firstly, qRT-PCR was performed to detect TTTY15 expression in GC tissue and cells. It was revealed that compared with para-cancerous tissues, TTTY15 expression in GC tissues was significantly increased (Figure 1(a)). In comparison with normal gastric mucosal epithelial cells GES-1, TTTY15 expression in GC cell lines was significantly increased (Figure 1(b)). After the patients were classified into high and low TTTY15 expression groups based on the average value of TTTY15 expression, chi-square test was performed, and it indicated that highly expressed TTTY15 was strongly associated with advanced TNM stage and poor tumor differentiation of the patients (Table 1). Subsequently, through searching Kaplan–Meier Plotter database, it was revealed that high TTTY15 expression was significantly associated with the short OS and DFS time of GC patients (Figure 1(c,d)).

**Table 1.** Correlation between clinicopathological features and TTTY15 expression in GC

Pathological parameters	Numbers (n = 59)	TTTY15 expression High (n = 29) Low (n = 30)		χ^2^	*p-*Value
Age (years)				0.871	0.351
< 60	33	18	15		
≥ 60	26	11	15		
Tumor size (cm)				2.034	0.154
< 5	32	13	19		
≥ 5	27	16	11		
TNM stage				9.246	0.002*
I–II	36	12	24		
III–IV	23	17	6		
Lymph node metastasis				3.003	0.083
No	25	9	16		
Yes	34	20	14		
Degree of differentiation				4.441	0.035*
Low, medium	39	23	16		
High	20	6	14		

*P < 0.05.

### 3.2. Effects of TTTY15 on GC cell proliferation, migration, invasion, apoptosis and epithelial–mesenchymal transition (EMT) process

**Figure 2.**
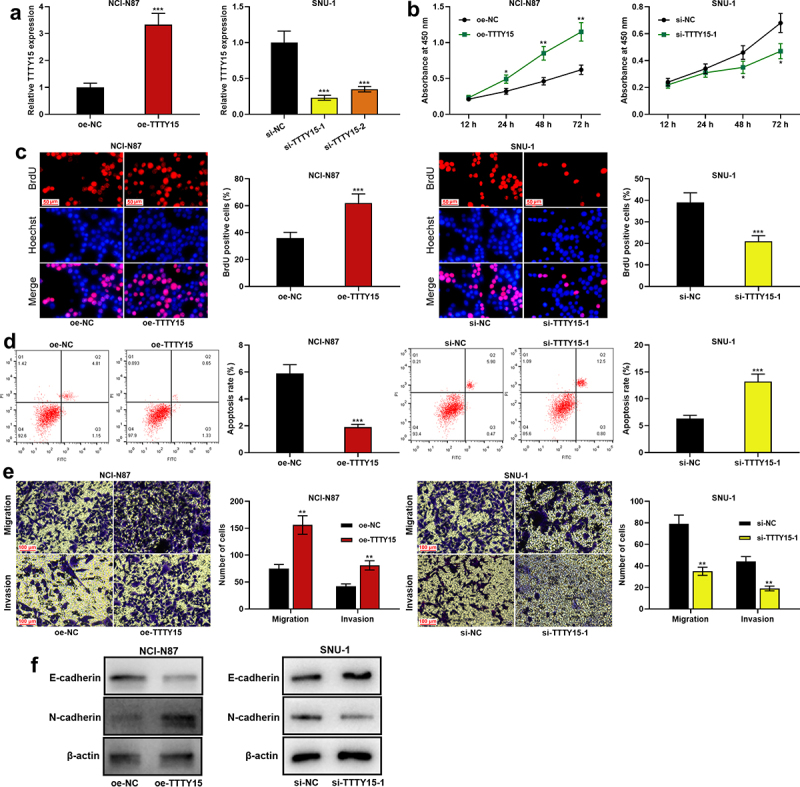
Effect of TTTY15 on GC cell proliferation, apoptosis, migration, invasion and EMT process. (a) The transfection efficiency of TTTY15 overexpression plasmid, si-TTTY15-1 and si-TTTY15-2 was detected by qRT-PCR. (b and c) CCK-8 method and BrdU assay were conducted for detecting the effects of TTTY15 overexpression and knockdown on the viability and proliferation of NCI-N87 and SUN-1 cells. (d) Flow cytometry assay was utilized for detecting the effects of TTTY15 overexpression and knockdown on the apoptosis of NCI-N87 and SUN-1 cells. (e) Transwell assays were performed to detect the effects of TTTY15 overexpression and knockdown on the migration and invasion of NCI-N87 and SUN-1 cells. (f) Western blot was carried out to detect the effects of TTTY15 overexpression and knockdown on the expression of EMT-related proteins in NCI-N87 and SUN-1 cells. All of the experiments were performed in triplicate. * *P* < 0.05, ** *P* < 0.01 and *** *P* < 0.001.

To further explore the biological functions of TTTY15 on GC cell proliferation, migration, invasion, apoptosis and EMT process, TTTY15 overexpression plasmid was transfected into NCI-N87 cells, and si-TTTY15-1 or si-TTTY15-2 was transfected into SUN-1 cells, which were confirmed by qRT-PCR to be successful (Figure 2(a)). CCK-8 and BrdU assays suggested that compared with the control group, TTTY15 overexpression significantly promoted NCI-N87 cell proliferation, while TTTY15 knockdown significantly inhibited SUN-1 cell proliferation (Figure 2(b,c)). Flow cytometry assay showed that compared with the control group, TTTY15 overexpression suppressed NCI-N87 cell apoptosis, whereas knocking down TTTY15 facilitated SUN-1 cell apoptosis (Figure 2(d)). Transwell assays revealed that as opposed to the control group, TTTY15 overexpression remarkably promoted NCI-N87 cell migration and invasion, while TTTY15 knockdown remarkably restrained SUN-1 cell migration and invasion (Figure 2(e)). Western blot was then performed for detecting the expression of EMT-related proteins in NCI-N87 and SUN-1 cells, and it was indicated that as against the control group, TTTY15 overexpression promoted N-cadherin protein expression and inhibited E-cadherin expression in NCI-N87 cells; knocking down TTTY15 promoted E-cadherin expression and suppressed N-cadherin protein expression in SUN-1 cells (Figure 2(f)).

### 3.3. TTTY15 directly targets let-7a-5p

**Figure 3.**
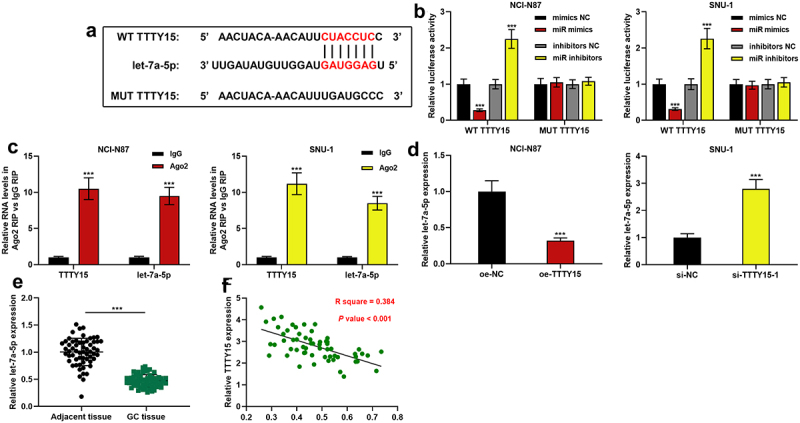
TTTY15 directly targets let-7a-5p. (a) The binding sites between TTTY15 and let-7a-5p were predicted using the online database StarBase. (b) WT TTTY15 and MUT TTTY15 luciferase reporter vectors were co-transfected with let-7a-5p mimics (miR mimics) or let-7a-5p inhibitors (miR inhibitors) into NCI-N87 and SUN-1 cells, respectively, and luciferase activity of the cells was used to validate the predicted binding site. (c) RIP assay was performed to validate the binding relationship between TTTY15 and let-7a-5p. (d) qRT-PCR was used to detect the effects of TTTY15 overexpression and knockdown on let-7a-5p expression in GC cells. (e) qRT-PCR was used to detect let-7a-5p expression in 59 pairs of GC tissues and para-cancerous tissues. (f) Pearson correlation analysis of the correlation between let-7a-5p and TTTY15 expressions in GC tissues. All of the experiments were performed in triplicate. *** *P* < 0.001.

To decipher the downstream mechanism of TTTY15, StarBase database (http://starbase.sysu.edu.cn/) was searched, and it showed that let-7a-5p was one of the potential functional target miRNAs of TTTY15 (Figure 3(a)). Subsequently, dual-luciferase reporter gene assay was performed to explore the interaction between TTTY15 and let-7a-5p. It was revealed that the transfection of let-7a-5p could suppress the luciferase activity of WT TTTY15, and the transfection of let-7a-5p inhibitor could increase the luciferase activity of WT TTTY15, whereas the transfection of miR mimics or miR inhibitors did not significantly affect the luciferase activity of MUT TTTY15 (Figure 3(b)). Furthermore, RIP assay validated that in comparison to IgG group, TTTY15 and let-7a-5p were significantly enriched in Ago2-containing microribonucleoproteins (Figure 3(c)). Next, qRT-PCR showed that TTTY15 overexpression inhibited let-7a-5p expression, while TTTY15 knockdown significantly promoted let-7a-5p expression (Figure 3(d)). Additionally, compared with para-tumorous tissues, let-7a-5p expression was remarkably decreased in GC tissues (Figure 3(e)), and TTTY15 and let-7a-5p expression levels were negatively correlated (Figure 3(f)).

### 3.4. Effects of TTTY15 and let-7a-5p on GC cell proliferation, migration, invasion, apoptosis and EMT process

**Figure 4.**
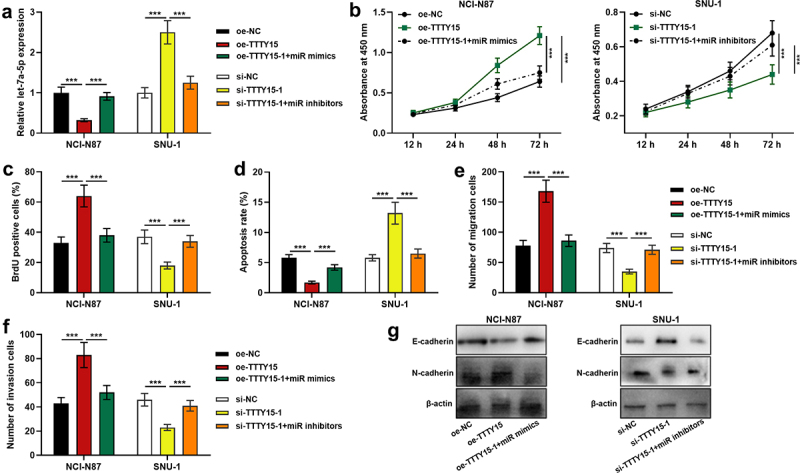
Effects of TTTY15/let-7a-5p axis on GC cell proliferation, apoptosis, migration and invasion. (a) qRT-PCR was used to detect the transfection efficiency of TTTY15 overexpression plasmids + miR mimics and si-TTTY15-1 + miR inhibitors. (b and c) CCK-8 method and BrdU assay were used to detect the effects of TTTY15 and let-7a-5p on the viability and proliferation of NCI-N87 and SUN-1cells. (d) Flow cytometry assay was used to detect the effects of TTTY15 and let-7a-5p on NCI-N87 and SUN-1 cell apoptosis. (e and f) Transwell assays were used to detect the effects of TTTY15 and let-7a-5p on the migration and invasion of NCI-N87 and SUN-1 cells. (g) Western blot was used to detect the effects of TTTY15 and let-7a-5p on the EMT process of NCI-N87 and SUN-1 cells. All of the experiments were performed in triplicate. ** *P* < 0.01 and *** *P* < 0.001.

To delve into the effects of TTTY15 and let-7a-5p on GC cell proliferation, apoptosis, migration and invasion, we co-transfected TTTY15 overexpression plasmids + let-7a-5p mimics into NCI-N87 cells, and si-TTTY15-1 + let-7a-5p inhibitors into SUN-1 cells, and the transfection was confirmed by qRT-PCR to be successful(Figure 4(a)). CCK-8 assay, BrdU assay, flow cytometry, Transwell assays and Western blot indicated that as against the control group, TTTY15 overexpression markedly facilitated cell proliferation, migration, invasion and EMT, and suppressed cell apoptosis, while the transfection of let-7a-5p reversed these effects (Figure 4(b–g)); knocking down TTTY15 remarkably restrained cell proliferation, migration, invasion and EMT, and induced cell apoptosis, whereas the the transfection of let-7a-5p counteracted these effects (Figure 4(b–g)).

### 3.5. TTTY15 activates the Wnt/β-catenin signaling pathway by regulating the let-7a-5p/Wnt1 axis

**Figure 5.**
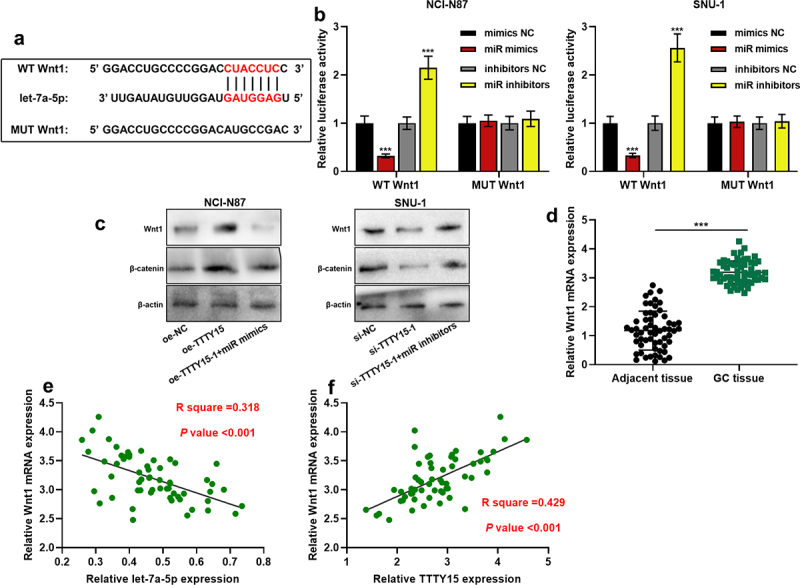
TTTY15 regulates the Wnt/β-catenin signaling pathway by adsorbing let-7a-5p (a) The online database TargetScan was used to predict the binding site between let-7a-5p and Wnt1 mRNA 3'UTR. (b) WT Wnt1 and MUT Wnt1 luciferase reporters were co-transfected with let-7a-5p mimics (miR mimics) or let-7a-5p inhibitors (miR inhibitors) into NCI-N87 and SUN-1 cells, respectively, and dual-luciferase reporter gene assay was used to detect the luciferase activity. (c) Western blot was used to detect the regulatory effects of TTTY15 and let-7a-5p on the protein expression of Wnt1 and β-catenin in NCI-N87 and SUN-1 cells. (d) qRT-PCR was used to detect Wnt1 expression in 59 pairs of GC tissues and adjacent tissues. (e and f) Pearson correlation analysis of the correlation between the expressions of Wnt1 and let-7a-5p, and the expressions of Wnt1 and TTTY15 in GC tissues. All of the experiments were performed in triplicate. *** *P* < 0.001.

To further clarify the downstream regulatory mechanism of let-7a-5p, the downstream targets of let-7a-5p were predicted using the TargetScan database (http://www.targetscan.org/), and it was found that Wnt1 was one of the downstream targets of let-7a-5p (Figure 5(a)). Dual-luciferase reporter gene assay revealed that the transfection of let-7a-5p mimics inhibited the luciferase activity of WT Wnt1, and the transfection of let-7a-5pinhibitors promoted the luciferase activity of WT Wnt1, whereas the transfection of mimics or inhibitors had no significant effect on the luciferase activity of MUT Wnt1 (Figure 5(b)). Western blotting indicated that TTTY15 overexpression increased Wnt1 and β-catenin protein expression, and the transfection of let-7a-5p mimics counteracted this effect; TTTY15 knockdown decreased the expressions of Wnt1 and β-catenin protein, while the transfection of let-7a-5p inhibitors reversed this effect (Figure 5(c)). It was also found through qRT-PCR that as opposed to para-cancerous tissues, Wnt1 mRNA was significantly highly expressed in GC tissues (Figure 5(d)), and let-7a-5p and Wnt1 mRNA expressions were negatively correlated, while TTTY15 and Wnt1 mRNA expressions were positively correlated (Figure 5(e,f)).

## 4. Discussion

LncRNAs can take part in modulating multiple biological processes, and they are also important regulators in cancer biology [27,28]. Previous studies have shown that TTTY15 is implicated in the regulation of a series of physiological and pathological processes. For example, knocking down TTTY15 reduces H_2_O_2_-induced cardiomyocyte injury via modulating the miR-98-5p/CRP axis [29]; TTTY15 can promote hypoxia-induced vascular endothelial cell injury by down-regulating miR-186-5p [30]. Importantly, TTTY15 is also involved in regulating the proliferation, differentiation, apoptosis and other biological processes of cancer cells [9,11,31]. Our previous study reports that TTTY15 up-regulates disheveled segment polarity protein 3 (DVL3) expression by adsorbing miR-29a-3p, thus promoting the malignancy of colorectal cancer cells [11]. Another study reports that TTTY15 promotes CDK6 and FN1 expressions by adsorbing let-7, therefore promoting PCa cell proliferation [9]. The above studies indicate that TTTY15 is an oncogenic lncRNA in these cancers. However, low TTTY15 expression is associated with an increase in the TNM stage of non-small cell lung cancer; TTTY15 overexpression targets and affects T-box transcription factor 4 (TBX4) expression via DNA (cytosine-5)-methyltransferase 3A (DNMT3A)-mediated regulation, thereby suppressing the cell cycle progression and metastasis of non-small cell lung cancer cells [31]. This study suggests that TTTY15 is a tumor suppressor in lung cancer. In the present study, for the first time, we demonstrated that TTTY15 expression was enhanced in GC tissues, and its high expression was associated with the patient’s poor prognosis; additionally, TTTY15 overexpression significantly facilitated the malignant biological behaviors of GC cells. Our data suggest that TTTY15 plays a cancer-promoting role in GC.

Known as a kind of short single-stranded non-coding RNA, microRNAs (miRNAs or miRs) are characterized by a length of 19–22 nucleotides, and mainly induce the translation inhibition or degradation of the target mRNAs through binding with the 3'UTR of the target mRNAs, thereby modulating gene expression at the post-transcriptional level [32], thus participating in various biological processes, including cell proliferation, differentiation, survival, metabolism, inflammation, migration and angiogenesis [33,34]. Hsa-let-7a-1 belongs to let-7 family, and hsa-let-7a-5p (MIMAT0000062) is one of the mature hsa-let-7a-1 transcripts [^33–35^]. Some studies have suggested that let-7a-5p represses the progression of GC [^33–35^]. For instance, it promotes GC cell autophagy by targeted down-regulation of Rictor expression to inhibit GC progression [33]; let-7a-5p blocks GC cell cycle progression and inhibits cell metastasis by suppressing RAB40C expression [34]; another study reports that let-7a-5p represses GC cell proliferation, migration and invasion via decreasing PKM2 expression [35]. In this work, it was revealed that let-7a-5p was a downstream target of TTTY15 and was negatively modulated by it. This is a new explanation for the mechanism of the abnormal expression of let-7a-5p in GC.

‘Cancer stem cell’ (CSC) theory believes that the tumorigenesis, metastatic potential and drug resistance of tumor depend on a small cell population with self-renewal capacity [36]. CSC can expand its number through symmetric division, or accelerate tumor growth through asymmetric division, and the existence of CSC in GC is considered to be the possible cause of GC tumorigenesis, progression, treatment resistance and poor prognosis [37,38]. In GC, the excessive activation of the Wnt/β-catenin signaling is also regarded as one of the typical features of CSC [39]. For example, tissue transglutaminase-1 (TGM1) can promote the stemness and chemotherapy resistance of GC cells by activating the Wnt/β-catenin pathway [14]; DVL3 participates in activating the Wnt/β-catenin pathway to enhance the CSC stemness of GC cells [40,41]. Noteworthily, let-7 family members are also key regulators of the Wnt/β-catenin signaling. In hepatocellular carcinoma, let-7a-5p directly targets Wnt1 to reduce the stemness of cancer cells [42]; and in breast cancer, let-7c inhibits the Wnt/β-catenin signaling activation to induce the asymmetric division of CSC [43]. Our study confirmed that TTTY15 activated the Wnt/β-catenin signaling via modulating the let-7a-5p/Wnt1 axis. Intriguingly, our previous study suggested that TTTY15 can positively regulate DVL3 expression, leading to activation of Wnt/β-catenin signaling pathway [11]. These demonstrations indicate that TTTY15 is a crucial regulator of Wnt/β-catenin signaling, which supports that TTTY15 promotes GC tumorigenesis in men.

## 5. Conclusion

To sum up, our study reports that the abnormal expression of male-specific lncRNA TTTY15 promotes GC progression via regulating let-7a-5p/Wnt1 pathway. This work may partly explain the difference in the incidence and prognosis of GC among men and women. However, the present study has some limitations. Firstly, the biological function of TTTY15 in GC requires validation in animal models. Secondly, the regulatory function of TTTY15 on the stemness of GC cells remains to be investigated in the following work.

## Data Availability

The data used to support the findings of this study are available from the corresponding author upon request.
